# Line-Field Optical Coherence Tomography as a tool for *In vitro* characterization of corneal biomechanics under physiological pressures

**DOI:** 10.1038/s41598-019-42789-4

**Published:** 2019-04-19

**Authors:** Ahmed Kazaili, Samuel Lawman, Brendan Geraghty, Ashkan Eliasy, Yalin Zheng, Yaochun Shen, Riaz Akhtar

**Affiliations:** 1 Department of Mechanical, Materials and Aerospace Engineering, School of Engineering, University of Liverpool, Liverpool, L69 3GH UK; 2 Department of Biomedical Engineering, College of Engineering, University of Babylon, Hillah, Iraq; 3 Department of Electrical Engineering and Electronics, University of Liverpool, Liverpool, L69 3GJ UK; 4 Institute of Ageing and Chronic Disease, University of Liverpool, Liverpool, L7 8TX UK

**Keywords:** Biomedical engineering, Imaging and sensing

## Abstract

There has been a lot of interest in accurately characterising corneal biomechanical properties under intraocular pressure (IOP) to help better understand ocular pathologies that are associated with elevated IOP. This study investigates the novel use of Line-Field Optical Coherence Tomography (LF-OCT) as an elastographic tool for accurately measuring mechanical properties of porcine corneas based on volumetric deformation following varying IOPs. A custom-built LF-OCT was used to measure geometrical and corneal surface displacement changes in porcine corneas under a range of IOPs, from 0–60 mmHg. Corneal thickness, elastic properties and hysteresis were calculated as a function of pressure. In addition, the effects of hydration were explored. We found that the elastic modulus increased in a linear fashion with IOP. Corneal thickness was found to reduce with IOP, decreasing 14% from 0 to 60 mmHg. Prolonged hydration in phosphate buffered saline (PBS) was found to significantly increase the elastic modulus and corneal hysteresis. Our study demonstrates that LF-OCT can be used to accurately measure the elastic properties based on volumetric deformation following physiological pressures. Furthermore, we show that prolonged hydration in PBS has a significant effect on the measured corneal properties.

## Introduction

A comprehensive understanding of the biomechanical properties of the cornea is important for many clinical applications, for example, accuracy of intraocular pressure (IOP) measurement^1–3^, treatment of keratoconus^4,5^, and corneal refractive surgery^6–8^. The biomechanical behaviour of the cornea is governed by its geometry and microstructure, which consists of integrated collagen type-I embedded in sub-ground matrix of proteoglycans. This structure exhibits nonlinear viscoelastic and anisotropic properties providing the biomechanical and geometrical characteristics that in turn contribute to the refractive power for satisfactory vision^4^. Corneal microstructure and geometry is also influenced by IOP, which should be in the range of 10–20 mmHg to maintain the normal shape and healthy function of the entire eye globe including the cornea^9^. It is for this reason that a number of studies have focussed on understanding how geometrical and biomechanical properties of the cornea are altered with elevated IOP^10–13^. With a better understanding of how corneal geometry and biomechanics are correlated with IOP, there is potential to gain new insight in to the diagnosis of some ocular conditions that in turn could lead to correcting ocular disorders and restoring sight^5,14–16^.

Studies that focus on corneal biomechanics under IOP typically utilise inflation (bulge) testing allowing corneal deformation to be assessed as a function of pressure^17–20^. Unlike conventional mechanical testing, inflation testing measures the biomechanical properties by expanding the entire cornea through a change in pressure whilst keeping the entire tissue intact^21–27^. In some studies, digital image correlation has been combined with inflation testing to provide spatially-resolved deformation mapping via of the cornea^12,19^. Other studies have measured the apical displacement-IOP curve for inflated corneas by using laser reflectometry^17,20,28^. In this approach, an artificial anterior chamber is used to hold the corneal samples and internal pressure is applied by using a PBS reservoir connected to the chamber. Scheimpflug imaging has also been used to capture the thickness of the cornea in inflation testing^29–31^. Although each of these corneal inflation studies demonstrate that elastic properties are altered with IOP, there are some limitations which must be considered. For example, the effect of optical and geometrical distortions of the cameras are underestimated and hence a number of correction factors are needed. These have been explained by Li *et al*.^32^ and also by Rosales and Marcos^33^. In addition, the methodologies employed in these studies do not allow real-time corneal geometrical changes to be captured with varying IOP. As a result, a number of assumptions have to be made about the elastic properties of the cornea.

Elastography measurements with optical coherence tomography (OCT) is a field which has emerged over the past two decades, and is termed optical coherence elastography (OCE)^34–51^. OCE has been developed and applied extensively to the cornea, as discussed in the comprehensive review by Larin and Sampson^52^. The non-invasive OCE technique employs optical coherence tomography (OCT) to detect the deformation of inflated corneas following applied external force on the cornea. These forces can be applied to the cornea in a contact (small indenter) or non-contact (e.g. air-pulse or acoustic wave) modalities. OCE techniques can also be used for biomechanical evaluation of the cornea following inflation. For example, a swept-source OCE system utilising an air-pulse has been used to test control and UV cross-linked porcine corneas of the same mechanical stiffness but under different IOPs^53^. This setup was successfully able to distinguish between the two groups, demonstrating the potential of such OCE methods.

However, OCE methods for assessing viscoelastic properties quantitatively are still at a very early stage^52^. One approach is to utilise static deformation through OCT and thereby exerting the cornea to a similar condition as with inflation test, as described earlier^17–20^. Ford *et al*.^37,47^ demonstrated such an approach for inflation utilising OCT, although the IOP range was limited and therefore the elastic modulus and hysteresis was not calculated. A recent study by Wang *et al*.^21^ effectively utilized optical coherence tomography (OCT)-based inflation testing for measuring the non-linear elastic behaviour of porcine corneas. This study makes an important contribution to the field of inflation testing via OCT because it bridges the gap between imaging via OCT and corneal biomechanical characterisation conducted with non-OCT based approaches as highlighted earlier. Specifically, they successfully demonstrated that a spectral domain OCT could be incorporated with inflation testing where the IOP is varied at different loading rates. They were able to observe the pressure-apex displacement relationship and calculate the apparent stiffness of the corneas using the axial deformation of the apex. Their study is more analogous to previous non-OCT studies^17–20^, where static deformation is captured for elasticity calculations rather than those in which dynamic deformation in response to a localized external force is monitored for elastic modulus calculation^34–40,42,43^. Elastic modulus values are intuitively comparable by both methods. However, corneal deformation (apex displacement) is not comparable due to the different force modalities. Although an important step forward in integrating OCT and corneal biomechanics, a limitation in the work of Wang *et al*. appears to be that the change of corneal thickness and the elastic modulus values following hydration in saline solution was not considered^21^. It is well-established that hydration has a significant effect on corneal geometry and biomechanical properties^31,41^. In addition, their work did not consider the role of corneal thickness in strain calculations, given that the approach they utilised is based on the assumption of axial deformation of the corneal apex only^22^.

In this study, we present a new inflation method utilising Line-Field OCT (LF-OCT) to measure the corneal geometrical parameters during the loading and unloading phases of corneal inflation in real-time. Our approach provides significant advantages over previous methods including a large scan size which gives sufficient resolution to accurately measure corneal apex geometrical changes including its displacement, real-time monitoring and high axial resolution. In addition, the resulting elastic properties and hysteresis are quantitatively correlated with hydration time.

## Materials and Methods

Eight fresh porcine eyes were obtained from a local abattoir shortly after slaughter. The pigs were aged from 5 to 6 months. The corneas, with a 2 mm scleral ring, were dissected and placed in a Barron Artificial Anterior Chamber (Katena Company, USA). An elevated reservoir of Phosphate Buffered Saline solution (PBS) (Sigma-Aldrich, Dorset, UK) was used to apply a hydraulic pressure to the posterior surface of the corneas, simulating intraocular pressures (IOP) of 0 to 60 mmHg. The pressure was controlled by the height of the reservoir and measured using an ABP series pressure sensor (Honeywell, NJ, USA). The corneas were measured at 0 mmHg and 2 mmHg, and then in 5 mmHg increments from 5 mmHg up to 60 mmHg during the loading phase. This sequence was then inverted for the unloading phases. The testing at each pressure step took 20 seconds, and time between pressure steps was 10 seconds. The experiments were carried out at room temperature (approximately 22.5 °C) and 45 ± 2.1% relative humidity, as determined with a humidity-temperature meter (OMEGA Engineering Ltd., Manchester, UK). One drop (60 µl) of PBS was applied on the external surface of the corneas (epithelium) every 4 min to maintain hydration. The testing was completed within 15 min after fixing the samples in the holder. The described experimental protocol was repeated every hour for four hours to detect the effects of corneal hydration on mechanical properties.

**Figure 1:**
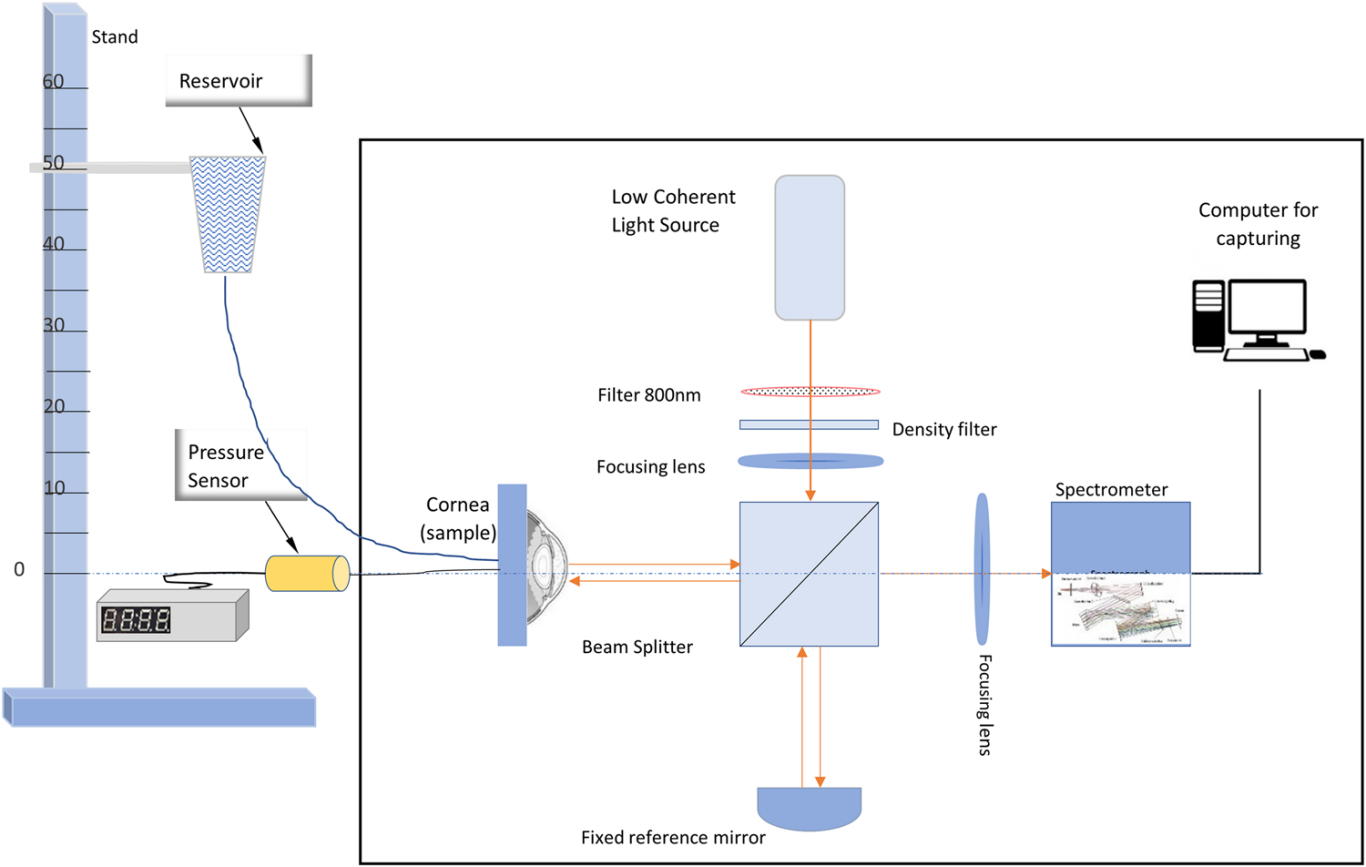
Schematic diagram for the combined corneal inflation and LF-OCT setup. The artificial anterior chamber was filled with PBS and connected to a reservoir which moved vertically up and down on a graded stand to control the pressure.

A LF-OCT system detailed elsewhere^54–56^ was used for this study. A schematic of the experimental setup is shown in Fig. 1. The system was setup with a CCD camera (iVac, Andor, UK), 2 Hz frame rate, 75 mm objective and 100 mm collection achromatic lenses. The commercial Czerny-Turner spectrograph (Shamrock 303i, Andor, UK) utilised with the system provides flexibility in the axial resolution by selection of the grating on the mechanical turret. In this study, a medium-resolution mode was selected by using a 600 lines/mm grating. This gave a larger axial range than at a higher axial resolution mode to facilitate monitoring of the corneal surfaces of the swelling porcine corneas while maintaining an axial resolution of approximately 3.5 μm in the corneal tissue. The signal to noise ratio (SNR) of the system was 79 dB, with a sensitivity of 93 dB and a roll off of 15 dB at 1mm^54^. For this study, the sensitivity of the device was sufficient to distinguish the surfaces of the corneas. The group refractive index of the fresh porcine cornea at the central wavelength of 800 nm was assumed to be 1.389 based on previous studies^57–60^. OCT images were analysed by using a semi-automated code written in MATLAB (Mathworks Company, Natick, Massachusetts, USA) to identify the boundaries of corneas using a segmentation technique. The corneal apex thickness, radius of curvature and displacement were measured with time at each pressure level. The corneal apex thickness was calculated and averaged for a cross-sectional image of the central 3 mm of the cornea. In our OCT system, the total thickness error/repeatability of approximately 2.2 µm was deemed acceptable. The adjustable microscale stage of sample arm and the flexibility of selecting the big axial depth (low-resolution mode 1200 lines/mm grating) were used to take the initial measurement of the height of the parabolic volume under the cornea (*H*_*o*_) at 2 mmHg.

### Calculation of Elastic Properties

**Figure 2:**
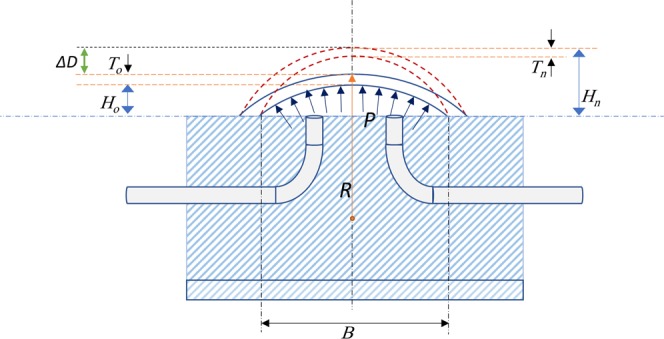
Schematic diagram showing a thin-walled sphere model for representing the cornea fixed in the holder (cornea-only model). *T*_*o*_ and *T*_*n*_: Apex corneal thickness (initial and current), *H*_*o*_ and *H*_*n*_: The height of parabolic volume under the cornea (initial and current), *B*: The base of the parabolic shape (*B* = 5.25 mm). *R*: Radius of curvature of the anterior surface of the corneal apex, *P*: Intraocular pressure, *ΔD* = *D−D*_*o*_, and *V*_o_ = (π/2)H_o_B^2^ (initial volume). In this model, a rigid sclera was assumed. The centre of corneal curvature was assumed to be fixed in the central axes of the cornea. Dashed arcs at the top of the figure represent the deformed (inflated) cornea.

The bulk elastic properties of the cornea were calculated by applying thin-walled sphere theory and Hooke’s law, since the thickness of the cornea is much smaller than the radius and the properties are measured at discrete pressure steps. The collected geometrical data was used to calculate the circumferential stress (*σ*) at each pressure level using equation (1)^61,62^:

1 $$\sigma =\frac{P.R}{2.T}$$

where *P* is the internal pressure, *R* is the radius of curvature and *T* is corneal apex thickness, see Fig. 2.

Figure 2 shows the model of thin-walled sphere theory and the needed assumptions. The volumetric strain (*ε*) was calculated from the geometrical variations of the cornea in response to IOP changes as shown equation (2). The initial displacement *D*_0_ and the interior apical height *H*_o_ were initially measured at 2 mmHg, where the cornea starts exhibiting curvature and stability of the corneal apex displacement^12^.

2 $$\varepsilon \,=\,\frac{{V}_{o}-V}{{V}_{o}}$$

The tangent elastic modulus (*E*_*c*_) was calculated by using equation (3)^62^:

3 $${E}_{c}=3(1-v)\frac{\sigma }{\varepsilon }$$

where *v* is Poisson’s ratio for the corneas. *v* was assumed to 0.40 based on a study that showed that the cornea behaves as a slightly compressible body^37^.

The stress-strain relationship was calculated for both loading and unloading phases. Corneal hysteresis was then calculated by measuring the area between the curves of the loading and unloading phases to show the cornea’s ability to absorb and dissipate energy.

### Statistical Analysis

All Statistical analysis was carried out in OriginPro 2016 version 9.3 (OriginLab, MA, USA). All data are expressed as mean values and standard deviation (mean ± standard deviation). The statistical significance of corneal thickness change of the samples over time at each IOP was calculated by using Wilcoxon signed ranks test. The statistical significance of elastic modulus and hysteresis changes with hydration time was also measured by the same method. P < 0.05 was considered as statistically significant for all tests.

## Results

**Figure 3:**
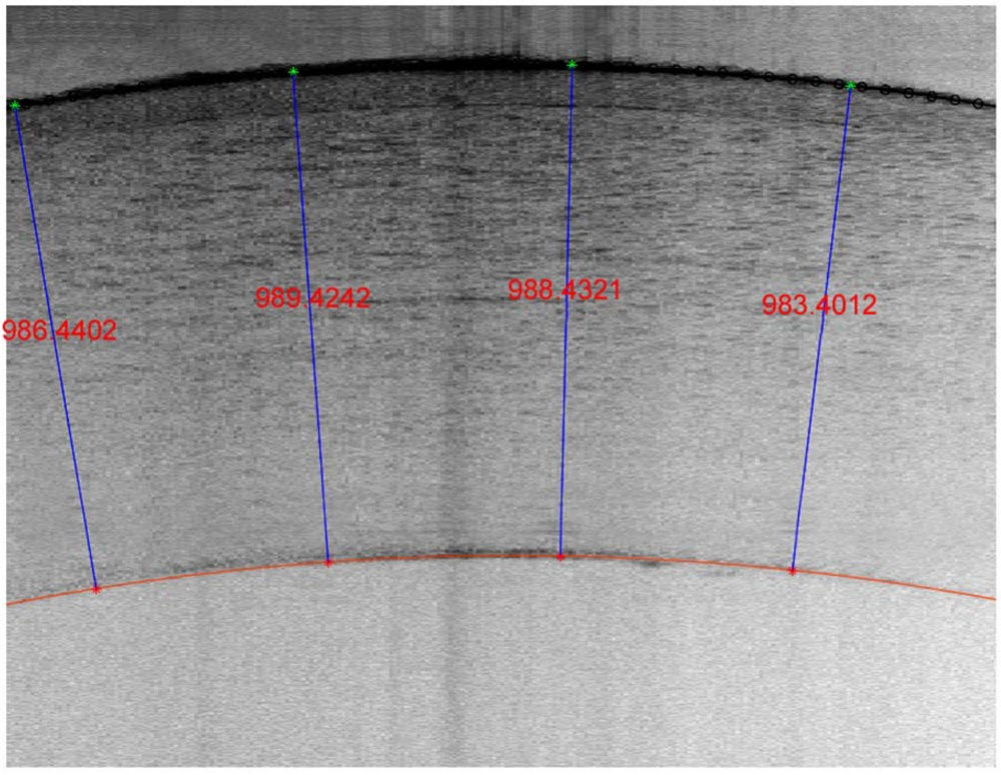
A typical LF-OCT image for a cornea at a pressure of 15 mmHg during the first loading phase (Time = 0 hour). The epithelium layer is clearly visible in the image and can be identified by the thin arc below the anterior surface of the cornea. The average thickness of this cornea was 986.9 µm.

Figure 3 shows an example LF-OCT image of a cornea inflated at 15 mmHg.

### Geometrical Variations

**Figure 4:**
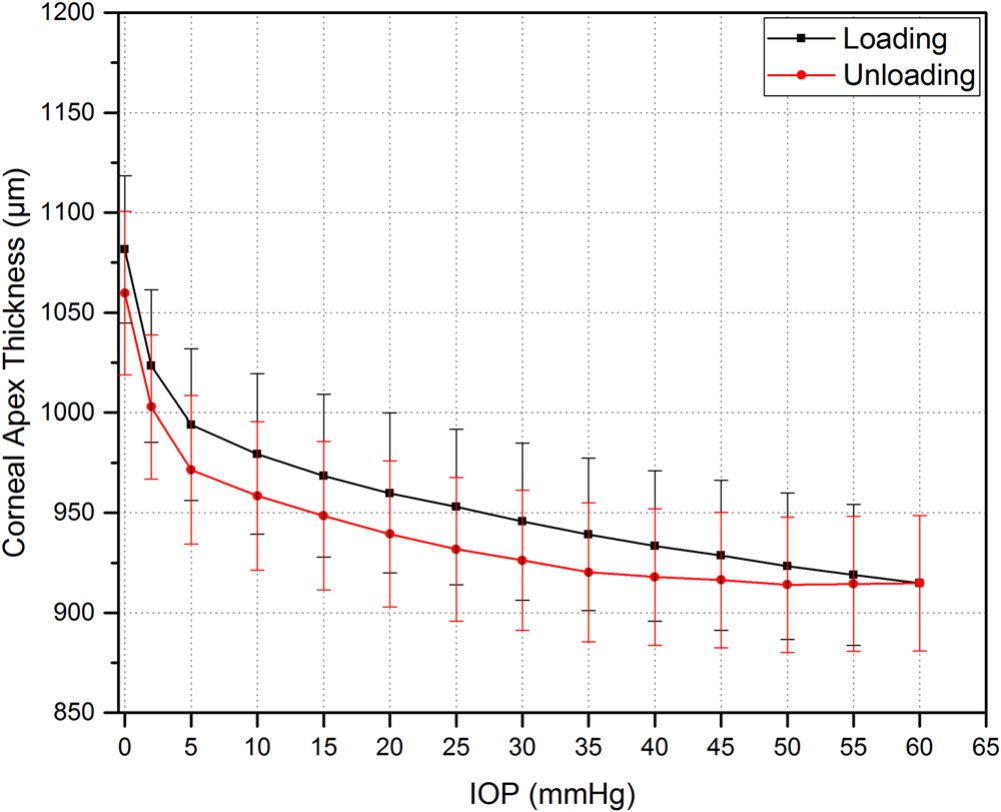
Mean thickness change of intact corneas (epithelium, stroma, and endothelium) with varying IOP. Errors bars represent standard deviation (n = 8 corneas).

Geometrical variations were detected by monitoring the corneal response to changes in IOP. Monitored parameters included cornea thickness, radius of curvature and displacement of the corneal apex. Figure 4 illustrates the thickness change with varying IOP during loading and unloading. Generally, the results show that corneal thickness is inversely proportional to increasing and decreasing internal pressure, and there is a significant difference in the corneal apex thickness between the loading and unloading phases (p ≤ 0.00024). It was noticed that corneas exhibited the expected curvature at 2 mmHg.

The best fit for the variation in corneal thickness during the loading phase follows a logarithmic equation which is shown in equation (4). However, there is approximately a linear relationship between the thickness of the cornea and the internal pressure of the loading phase from 15 to 60 mmHg. At high IOPs, the average reduction in the apex corneal thickness every 5 mmHg was approximately 1.07%.

4 $$T(p)=1085.5-57.3\,{P}^{0.265}$$

where *T(p)* is the thickness of the corneal apex in µm at specific IOP. *P* is the *IOP* measured in mmHg.

The percentage reduction in thickness at low internal pressures is significantly greater than that at high internal pressures. In the loading phase, the total change in corneal apex thickness reduced around 14% after the IOP was increased from 0 mmHg to 60 mmHg, which meant that the thickness reduced from 1081 ± 55 µm to 914 ± 33 µm. It was noticed that after the unloading phase finished at 0 mmHg, the corneal thickness did not immediately return to its original thickness i.e. at the point where the loading phase began. However, it was noted that the thickness did eventually recover after approximately 5 min.

**Figure 5:**
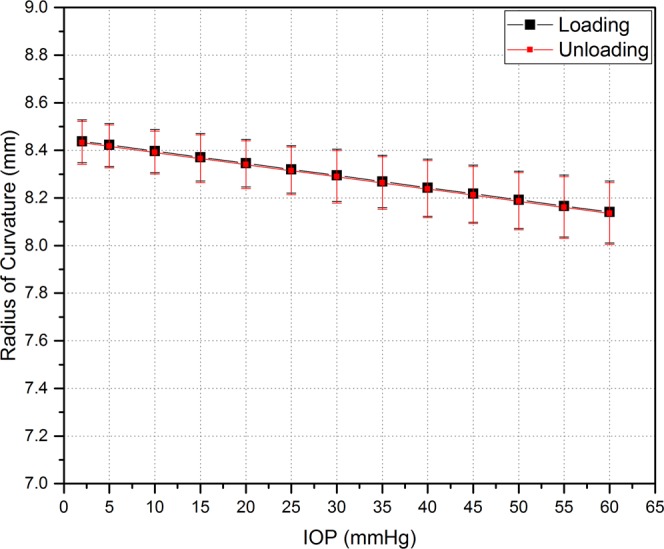
Radius of curvature of the corneal apex for corneas which were fixed in an artificial holder. Data is shown for 2 mmHg onwards. Error bars represent standard deviation (n = 8 corneas).

The radius of the corneal apex curvature decreased slightly when the IOP increased as shown in Fig. 5. At each specific IOP, the radius of corneal apex curvature in the loading phase was not significantly different than that in the unloading phase (p = 0.945). A linear relationship was observed between radius of corneal apex curvature and IOP, equation (5).

5 $$R(P)=8.448-0.005\times P$$

**Figure 6:**
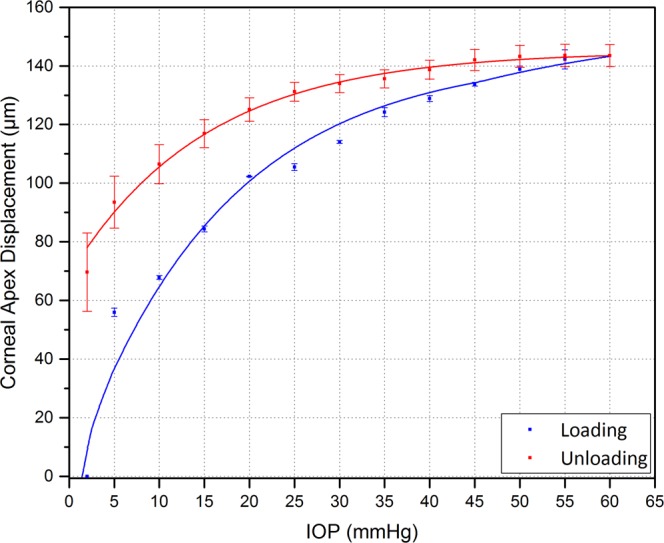
Corneal apex displacement with varying IOP loading and unloading. Vertical bars represent the standard deviation (n = 8 corneas).

The displacement of the corneal apex for the first loading and unloading phases is shown in Fig. 6. An exponential relationship was observed for both loading and unloading phases. The maximum corneal apex displacement was 143 ± 3.78 µm away from the initial position at 2 mmHg. The rate of change in the corneal apex displacement was greater at pressure levels below 20 mmHg.

### Mechanical Properties

**Figure 7:**
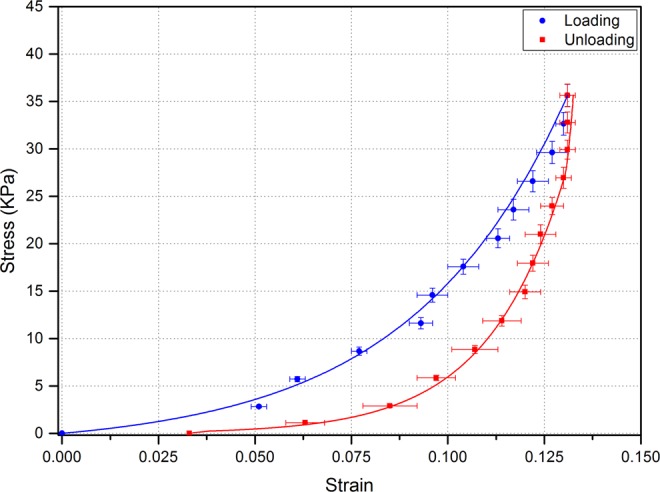
Corneal apex stress – strain curves obtained from the first loading and unloading phases (n = 8 corneas).

Elastic properties were calculated by the use of geometrical changes to IOP change and applying equations 1, 2 and 3. Corneal apex stress-strain curve for the first loading and unloading phases is shown in Fig. 7. Corneal apex hysteresis from the first loading and unloading phases was 10.5 ± 0.6 mmHg (1.4 ± 0.08 kPa) for the fresh corneas.

**Figure 8:**
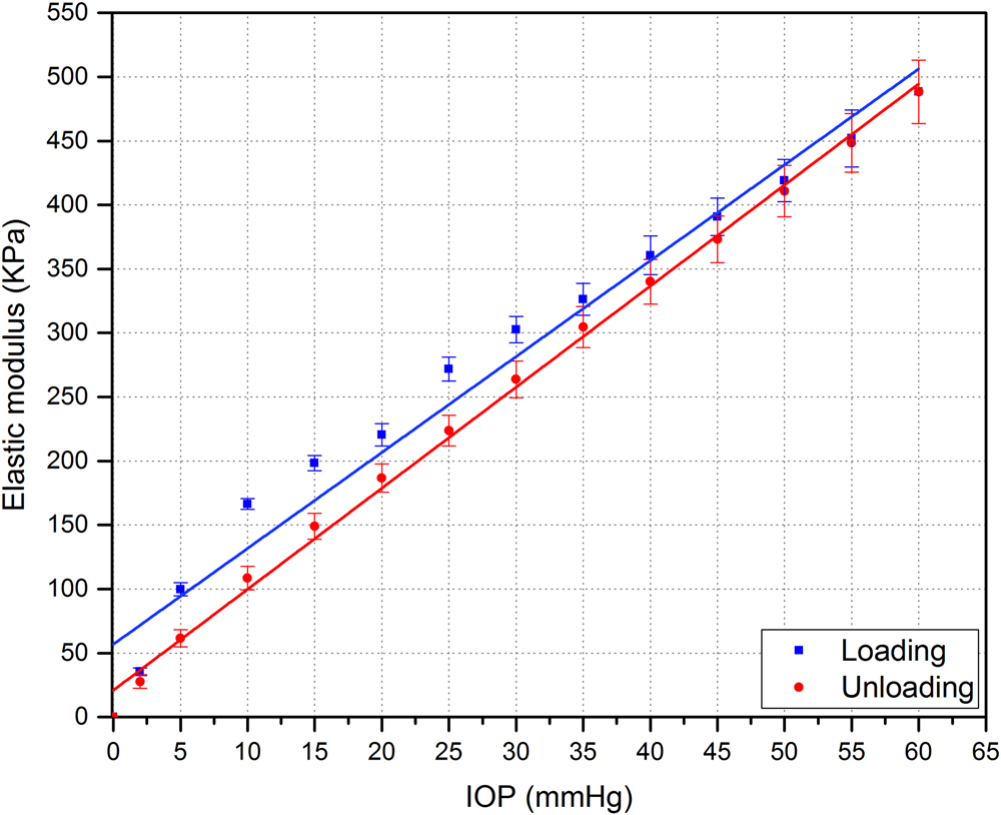
Elastic modulus of corneal apex obtained from the first loading and unloading phases (n = 8 corneas). There was significant difference between the elastic modulus of loading and unloading phases with varying IOP (tested by Wilcoxon signed ranks test, p = 0.0076).

The elastic  modulus of the corneal apex during the first loading and unloading phases is shown in Fig. 8 where (approximately) a linear relationship can be seen. However, in the loading phase, a non-linear relationship between the elastic modulus and IOP was noticed at pressure levels below 15 mmHg. There was a slight difference between the elastic modulus determined during the loading and unloading phases, which decreased as the IOP increased.

### Hydration Effects

**Figure 9:**
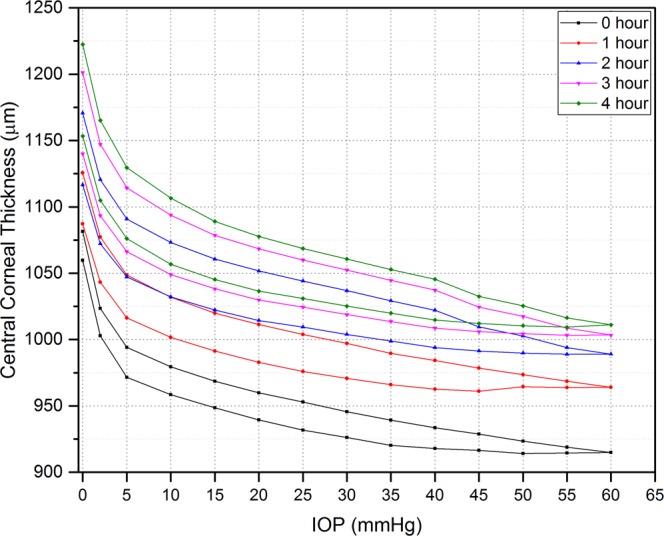
Plot showing corneal thickness change of the corneas with varying IOP for 0–4 hours. Each loop represents the corneal thickness during loading and unloading phases. Standard deviation values were excluded to allow the trends to be more discernible (n = 8 corneas). There was a significant difference between central corneal thickness of each cycle over time (Wilcoxon signed ranks test, p < 0.00012).

Figure 9 shows that corneal thickness during the loading and unloading phases was affected by two parameters, namely IOP and hydration time. It can be seen that the thickness of the corneas significantly increased over the hydration time (p ≤ 0.0012). Corneal thickness significantly increased by approximately 6% after the first hour of hydration in PBS. The maximum thickness recorded at 0 mmHg was 1222.47 ± 63.79 µm after four hours of hydration, which is about 14% more than that measured at the initial state. Figure 9 also shows that the area between the loading and unloading curves increased every hour, which indicates a change in viscous properties of the corneas. No visual damage to the corneas was detected during the repetitive measurements cycles.

**Figure 10:**
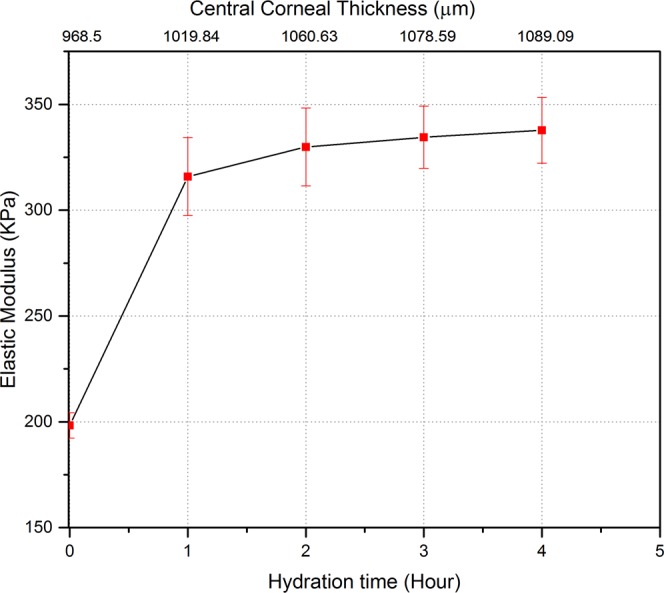
The change of elastic modulus during the loading phase with hydration time is correlated with central corneal thickness shown for IOP of 15 mmHg. The top x-axis represents the central corneal thickness at the corresponding hydration time (bottom x-axis) at 15 mmHg. Error bars represent standard deviation (n = 8 corneas). There was a significant difference between elastic modulus values between 0 hour and first hour of hydration, p ≤ 0.0026. Elastic modulus become relatively stable (slight increase) after the first hour of hydration.

The results also show that hydration can affect not only the thickness but also the mechanical properties of the corneas. An increase in the elastic modulus was observed due to geometrical changes of the samples, mainly thickness and corneal apex displacement. Figure 10 shows the increase in elastic modulus of the corneas at 15 mmHg with hydration time. The elastic modulus of hydrated corneas after 1 hour was significantly increased by approximately 52% relative to the initial elastic modulus when the corneas were almost fresh (zero hours). Elastic modulus of the corneas increased from 198.25 ± 5.97 KPa at 0 hour to 315.84 ± 18.4 KPa after 1 hour. Further, the elastic modulus of the corneas was found to significantly increase around 2% each hour after the first hour of hydration (P ≤ 0.0415).

**Figure 11:**
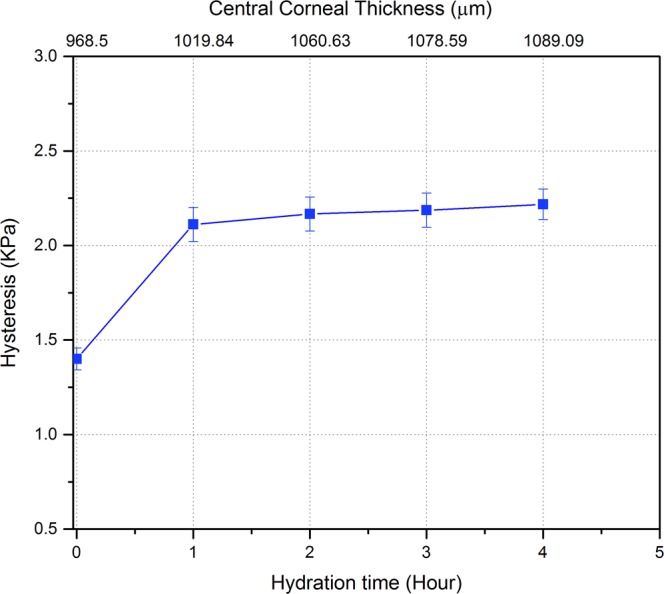
Corneal hysteresis and hydration time relationship (n = 8 corneas). The top x-axis represents the central corneal thickness at the corresponding hydration time (bottom x-axis) at 15 mmHg. There was a significant difference between hysteresis values over time between 0 hour and first hour of hydration, p ≤ 0.0078. Hysteresis became relatively stable (slight increase) after the first hour of hydration.

An increase in hysteresis was also detected with increasing hydration time. The hysteresis in hydrated corneas after 1 hour significantly increased by about 50% relative to that at the initial state; from 1.4 ± 0.08 KPa (10.5 ± 0.6 mmHg) to 2.1 ± 0.12 KPa (15.8 ± 0.9 mmHg) after 1 hour in PBS. Figure 11 shows the relation between hysteresis and hydration time. Hysteresis was found to significantly increase by around 2.7% from the first hour to the second hour of hydration; this percentage gradually decreased by 0.75% each hour (P ≤ 0.0336).

## Discussion

This study aimed to investigate the biomechanical properties of the porcine cornea using LF-OCT with application as a corneal inflation test method. The significant advantage of our approach is that it enables real-time LF-OCT to be used for monitoring corneal geometrical changes during loading and unloading cycles for four hours. A recent study used a spectral domain OCT for corneal inflation tests to obtain elastic deformation^21^. Although this experiment demonstrated a real-time method for static deformation for inflated corneas, the authors underestimated the effects of hydration on corneal thickness change after obtaining post-mortem corneas. This drawback can lead to inaccurate quantitative elastic deformation and unrealistic viscoelastic behaviour as stated previously^31,41^. In addition, the role of corneal thickness in strain calculations was not considered since it is based on the assumption of axial deformation of corneal apex only with no change in volume of corneal material during applied forces.

Utilising the advantages of LF-OCT system, real-time monitoring of geometrical changes was carried out with an axial resolution of approximately 3.5 μm in corneal tissue. This high-resolution monitoring equipment enabled us to obtain high-resolution cross-sectional images of the corneas that clearly showed the boundary between the epithelium and stromal layer (see Fig. 3). Therefore, the LF-OCT system can be considered as a superior imaging modality for monitoring the corneas during inflation testing as it provides both real-time and high-resolution data in a non-destructive manner. In addition, the utilized LF-OCT was more flexible than off the shelf commercial or research swept source systems as we have a motorised spectrograph and thus we were able to select the axial resolution and image depth range suitable for the task in hand. A similar LF-OCT system but with a lower axial resolution was demonstrated by Yasuno *et al*., in which three-dimensional OCT was built for *in vivo* dermatological investigation^63^.

The model in this study is based on a basic inflation model proposed by Anderson *et al*.^22^ and further development with the use of the approach that was proposed in the literature^24^ to obtain volumetric strain and tangent elastic modulus. The main limitation of this model is the assumption of zero scleral deformation; where, corneal deformation is recorded only. The cornea and sclera are flexibly connected in the native state, therefore, a whole-eye model including both the cornea and sclera would provide a better demonstration of *in-vivo* conditions^12,14,24^. However, the whole-eye model cannot accurately represent the native condition because the eye has an anisotropic structure. Hence, the sclera deformation is different than corneal deformation at the same IOP due to histological differences. Therefore, the corneal deformation in a whole-eye model will be affected by scleral deformation, and therefore complicated analysis is required to measure corneal behaviour^22^. The whole-eye model can better describe the ocular deformation as a whole structure instead of corneal deformation^14^. In the native state, the eye orbit and surrounding orbital tissues hold the eye ball in place; this acts to limit sclera deformation and the cornea deforms differently due to the constraints not being the same^22^. In addition, the whole-eye model is more complicated to maintain a steady IOP and is expensive to run^23^.

We have shown that the radius of the corneal apex decreases slightly with increasing IOP. This trend is similar to that seen in comparable studies^24,62,64,65^. In reality, the radius of the whole eye, both the cornea and the sclera, would be expected to slightly increase with increasing internal pressure. Unlike the cornea-only model used, the radius of corneal apex was slightly decreased because the free ends of the cornea were mechanically fixed and only its apex was free to deform (Fig. 2). Consequently, this fixation of the corneas at the limbus could exhibit a non-physiological boundary condition affecting measurements of corneal apex radius during inflation.

Our calculations of elastic behaviour depend on a four-dimension matrix of variables (*P, T, R* and *D*) which were varied with time for hydration and hysteresis assessment. The cycle of IOP change was set from 0 to 60 mmHg under which the porcine corneas were monitored for geometrical variations. Geometrical variations were utilized to extract the elastic behaviour during the loading and unloading cycles which was started from 2 mmHg in this study. Other studies have started from different IOPs, 10 mmHg^21,30^ and 5 mmHg^66^, depending on the linearity of the IOP-Apex corneal displacement curve. However, we decided to start from 2 mmHg for calculation of the elastic behaviour because we observed that corneas were sufficiently inflated at that minimal internal pressure (to remove the initial crimps from the corneal surface), in addition to exhibiting stable geometrical properties and corneal apex displacement. The experiments were performed in the IOP range of 0–60 mmHg and no visual damage was observed. Our observation and decision was in agreement with recent studies^12,28^.

Corneal thickness is an important parameter that can influence the accuracy of the IOP measurements^67–69^. Therefore, corneal thickness is often measured optically to obtain accurate measurements^40,70^. In this study, our mean corneal apex thickness measurement was 980 ± 13.5 µm at 15 mmHg. This is comparable to the thickness of 967 ± 76.5 µm at 15 mmHg, which has been reported by other studies that have utilised optical measurements^25,31,40^. The values also match *in-vivo* and *in-vitro* studies in which an ultrasound pachymeter was used for the measurements^71,72^. However, our value is around 30% lower than an *in-vivo* study in which a pachymeter was used for the measurements without considering the effects of IOP on the thickness measurement^73^. The trend of corneal thickness reduction and compression due to IOP has been well-described by other researchers^25,37,74^. The variations in absolute values of corneal apex thickness reported in the literature can be attributed to many factors including the age of the pigs and hydration method, but the testing instrument and method used are likely to be most significant factors^73,75^.

Corneal apex displacement curves are clearly non-linear throughout the whole IOP range. However, the slope varies slightly at high IOP (>20 mmHg). This non-linear behaviour demonstrates that the corneas have an average low stiffness at low IOP, and then the stiffness rises as the IOP increases. At low IOPs, the collagen fibrils are not taut, and the mechanical response is mainly dominated by the extracellular matrix of the stromal layer. At high IOPs, collagen fibrils lead the mechanical response. This non-linear response, i.e. change of slope rate in corneal apex displacement, has also been reported by other researchers^12,21,22,37^. Our values for corneal apex displacement with IOP for the porcine corneas are close to those reported by Whitford *et al*.^12^ and Wang *et al*.^21^.

The stress-strain relationship exhibits the expected non-linear mechanical behaviour. In the first cycle of the loading and unloading curves, no statistical significance was observed at low IOPs. This non-linear behaviour occurred due to the pre-conditioning phase of the cornea where the collagen fibrils are not aligned. Quinn and Winkelstein^76^ stated that there is a strong relationship between alignment changes of collagen fibrils and the mechanical response during pre-conditioning as fibrils start to change direction in response to the main deformation point. The porcine corneas tested in this study exhibited a typical viscoelastic response which is expressed by the hysteresis value measured from the area between the loading-unloading phases of the stress-strain curve. The initial hysteresis value for fresh porcine corneas, 10.5 ± 0.6 mmHg, is very close to the human cornea hysteresis measured *in vivo* by the Ocular Response Analyser^77^. The value of hysteresis can be affected by corneal pathology, for example it is low in keratoconic corneas^78^. It can also be influenced by the natural aging process^5,79^.

We found an approximately linear relationship between the elastic modulus and IOP, at IOPs greater than 20 mmHg. The values of elastic modulus are mostly non-linear at low IOPs. To the best of our knowledge, no previous study has reported the elastic modulus values of porcine corneas based on a similar method. We found that our elastic modulus results are comparable to a study conducted by Asejczyk-Widlicka and Pierscionek^24^, in which high-resolution digital cameras were used to monitor corneal profile changes in response to IOP. In addition, our elastic modulus values are slightly higher as compared to a study conducted by Singh *et al*.^40^. They reported that the elastic modulus was 14.7 KPa at 15 mmHg, when iOCE was used to assess the effects of UV-A/riboflavin corneal collagen crosslinking (CXL) on the mechanical anisotropy of *in situ* porcine corneas at varying IOPs. This difference could be related to the different method used and also due to the hydration effect on corneas. However, our study is comparable in the observed trend of increasing elastic modulus with decreasing CCT due to high IOP, as was reported in the same study^40^. Reporting elastic modulus of porcine corneas will help to identify mechanical properties and provide a basis for comparison with treated or unhealthy corneas. One limitation of our approach is that the elasticity calculation assumes that the corneal samples have an entirely homogenous thickness. In reality, there is an uneven increase in corneal thickness from central area toward the peripheral area^25^. This can lead to a slight inaccuracy in the calculation of volumetric strain since the thickness change of corneal apex (*ΔT*) is included in the calculation of the volumetric strain. Therefore, building a model to involve regional variation of corneal thickness will help to increase the accuracy of biomechanical properties calculations.

We also examined the effect of hydration on the biomechanical and geometrical properties of the corneas. Corneal thickness significantly increased after one hour. Since the pressure remained at 0 mmHg between tests, the thickness increase can be attributed to corneal swelling due to hydration. Corneal swelling likely occurred due to the osmolarity difference between the corneal tissue and PBS. The swelling gradually increased in subsequent test cycles indicating water quantity in the tissue is approaching the equilibrium state of the osmolarity difference. The area between the curves in each cycle gradually increased and hence there was a hysteresis change with each cycle. In the hydrated corneas, the elastic modulus significantly increased after one hour. After 4 hours of hydration, elastic modulus was substantially higher as compared to fresh corneas suggesting that this was due to the swelling (thickness increase). This relation proves that corneal thickness variations can affect the stiffness and then realistic IOP measurements. This is thought to be related to geometrical changes in the extracellular matrix and in particular collagen fibril properties due to swelling. These changes may lead to modifications in material density and molecular spacing, which will affect the biomechanical response. Therefore, corneal thickness should be maintained by appropriate hydration media which can help stabilise corneal elasticity. This finding was also proposed by Kling and Marcos^31^ where they preserved corneas in Dextran solution and found that they were less stiff than those in Optisol (tissue culture media). They also compared corneal thickness preserved in varying storage media, and studied the effect of hydration on porcine corneas. In addition, Dias and Ziebarth reported that the more swollen corneal samples (higher hydration) were stiffer (greater elastic modulus)^80^. They used AFM to examine the impact of corneal hydration on corneal elasticity of *ex-vivo* samples. On the other hand, Hatami-Marbini and Etebu reported contradictory results to the trend found within our study; where they showed that elastic modulus decreases with increasing hydration^81^. They used a rheometry to measure elastic response of corneas immersed in 0.9% NaCl solution. The difference may be related to the differences in characterization method, for example the method used by Hatami-Marbini and Etebu measures a shear response rather than an elastic response as in our study.

We also showed hysteresis variations with hydration time. Corneal hysteresis (the area between the loading and unloading phase of stress-strain curve) increased by 59.3% after 4 hours of hydration that made significant changes in corneal thickness. Our data shows that hysteresis increases as the central corneal thickness increases due to hydration. Since corneal thickness increases are associated with elastic modulus increases^36,41^, hysteresis may increase with high elastic modulus corneas. Although, there are limited studies in the literature which have examined corneal hysteresis as a function of hydration. One previous study has shown that the higher the concentration of Dextran, the greater the hysteresis observed^31^. In that study, hysteresis was defined as the amount of remaining corneal deformation after a cycle of increased/decreased IOP variation, which differs from our approach. Interestingly, the slightly swollen corneas in Optisol-GS for 24 hours showed increased hysteresis, which is in the agreement with our finding.

The approach used in this study is of clinical relevance. For example, keratoconic corneas are characterised by reduced localized thickness with a lower elastic modulus and hysteresis^4^. Furthermore, low corneal hysteresis and thin central corneal thickness are associated with glaucoma damage^82^. In addition, the findings can help in providing better models for calculating IOP in stiff corneas, and enhance the ability to accurately diagnose many other ocular diseases. Moreover, we have shown that corneal hydration has a significant influence on the biomechanical response of the cornea and the type of hydrating solution may have significant effects on the biomechanical response^31^. Therefore, geometrical changes due to hydration of corneal samples should be addressed to increase the accuracy of models and improve clinical practice.

In conclusion, we have developed an LF-OCT system for corneal inflation testing to provide better assessment for the corneal biomechanical behaviour. We have validated our data by comparing the results with other studies that used the same parameters. The results show that the biomechanical properties of the cornea can be influenced by many factors such as IOP, corneal thickness and hydration. Over the loading phase, corneal thickness decreases as the IOP increases. Elastic modulus increases as the IOP increases. Finally, the corneas showed a non-linear increase of elastic modulus, corneal thickness and hysteresis as the hydration time increased. Our method may help build better numerical and mechanical models and thereby help better understand corneal biomechanics. In addition, our study provides information that might help avoid complications in corneal surgical practice which may occur due to inaccurate estimation of the real IOP and corneal thickness.

## Data Availability

The datasets generated during and/or analysed during the current study are available from the corresponding author on reasonable request.
